# Effects of Cold-Pressing and Hydrodistillation on the Active Non-volatile Components in Lemon Essential Oil and the Effects of the Resulting Oils on Aging-Related Oxidative Stress in Mice

**DOI:** 10.3389/fnut.2021.689094

**Published:** 2021-06-14

**Authors:** Guijie Li, Sha Xiang, Yanni Pan, Xingyao Long, Yujiao Cheng, Leng Han, Xin Zhao

**Affiliations:** ^1^National Citrus Engineering Research Center, Citrus Research Institute, Southwest University, Chongqing, China; ^2^Chongqing Collaborative Innovation Center for Functional Food, Chongqing Engineering Research Center of Functional Food, Chongqing Engineering Laboratory for Research and Development of Functional Food, Chongqing University of Education, Chongqing, China; ^3^National Citrus Engineering Research Center, Chongqing, China; ^4^Department of Dermatology, Xinqiao Hospital, Army Medical University, Chongqing, China

**Keywords:** cold-expression, furocoumarins, coumarins, antioxidant, oxidative stress

## Abstract

The aim of this study was to analyze the non-volatile composition and antioxidant differences of lemon essential oils (LEOs) obtained by cold-pressing vs. hydrodistillation. Pathological observations showed that LEO effectively inhibited liver injury caused by oxidative stress, and CPLEO was more effective than HDLEO. CPLEO increased serum T-AOC, SOD, GSH, and GSH-Px levels while decreasing NO, COX-2, IL-6, IL-1β, IFN-γ, and TNF-α levels in mice with oxidative damage. The effects of CPLEO were stronger than those of HDLEO and similar to those of vitamin C. CPLEO upregulated mRNA and protein expressions of Cu/Zn-SOD, Mn-SOD, CAT, HO-1, Nrf2, and NQO1 while downregulating nNOS, iNOS, IL-1β, COX-2, TNF-α, and NF-κB mRNA expression and nNOS, eNOS, iNOS, and COX-2 protein expression in mice with oxidative damage. The results demonstrate that LEO has good antioxidant effects and that CPLEO has a better antioxidant effect than HDLEO as it retains more active non-volatile substances.

## Introduction

Lemon is an acidic fruit that is consumed by humans and is also one of the most medicinal fruits in the world. It is rich in vitamin C (Vc), calcium, phosphorus, iron, vitamin B1, vitamin B2, etc. Lemon prevents and eliminates skin pigmentation, improves vision and dark adaptability, reduces fatigue, and elicits other effects that are very beneficial to the human body (1). Lemon essential oil made from lemon peel retains and concentrates some of the aromatic and active substances in the peel and thus retains and enhances some of the biological activity of lemon peel (2). Studies have shown that lemon essential oil can improve circulatory system function; for example, it can promote blood circulation, reduce blood pressure and stop nosebleeds. It can also strengthen the immune system; improve digestive system function; decompose fat; treat dyspepsia and constipation; soothe headaches, including migraines; and help to treat arthritis and rheumatism by combining with

acidic substances in the body (3–5). Furthermore, lemon essential oil helps to clear acne, cleanse oily skin and hair and remove dead skin cells (6). In addition, the fresh aroma of lemon essential oil can refresh the mind and spirit, relieve irritability and purify the air (7).

Plant essential oils have direct and indirect antioxidant effects. In their antioxidant responses, some active components of antioxidants can react with excessive free radicals, reduce free radicals and maintain oxidation balance in the body (8). Antioxidants such as catalase and metal-chelating agents can prevent the formation of free radicals during the initial phase. In the propagation stage, antioxidants can react with peroxy radicals faster than the cyanidation matrix to produce relatively stable substances and slow or block the chain reaction (9). In indirect antioxidation refers, the beneficial substances do not directly participate in the process of antioxidation but rather play their roles by protecting the oxidation matrix or improving physiological antioxidant defense (10). The antioxidant activity of plant essential oils depends mainly on the abundant phenolic components of the oils, and the action pathways of the oils include scavenging of free radicals, chelation with metal ions, inhibition of cell membrane lipid peroxidation and regulation of antioxidant enzymes (11).

Lemon essential oil has been demonstrated to contain a variety of chemical components with strong antioxidant effects. However, maximum retention of the active components in lemons requires the selection of appropriate extraction methods for the essential oil. The common methods include cold-pressing, hydrodistillation, solvent extraction, molecular distillation, microencapsulated aqueous two-phase extraction and supercritical fluid extraction. The simplest method is hydrodistillation, but cold-pressing is currently commonly used in industrial settings (12, 13). Studies have shown that the cold-pressing method can retain the effective components of the original plants, and this method is economical and practical. However, there is a lack of relevant research on the component differences caused by different extraction methods and on the different antioxidant effects of the resulting oils (14). Vitamin C is an acid hexose derivative, which is enol caproic acid lactone. Alcoholic hydroxyl is easy to be oxidized into carbonyl, so it has oxidation state. VC plays the role of electron transfer in the body through the tautomerism of oxidation state and reduction state. Vitamin C is usually used as an antioxidant, because it is easy to be oxidized. It can replace other substances to be oxidized first, so as to protect other substances from oxidation. It is a safe antioxidant (15). Therefore, vitamin C was selected as a positive control. In this study, the non-volatile components of lemon essential oil extracted by cold-pressing and hydrodistillation were compared. The antioxidant effects of the two kinds of lemon essential oil were assessed in mice, and the effects of the differences in essential oil components caused by the different extraction methods on the antioxidant mechanisms were clarified. The commonly used antioxidant Vc was selected as the positive control. Olive oil, which was used as the solvent for the lemon essential oil extracted by the two methods and which has certain antioxidant effects of its own, was used as the negative control.

## Materials and Methods

### Samples, Standards, and Solvents

Fresh mature Eureka lemon fruits were harvested from a normally managed orchard of the National Citrus Germplasm Repository (106°43′ E, 29°83′ N; altitude 241 m), Chongqing, China. Cold-pressed lemon essential oil (CPLEO) was obtained by using a method described in our previous study (16). Hydrodistilled lemon essential oil (HDLEO) was obtained by using an improved Clevenger apparatus with a second condenser as described by Chen et al. (17). Briefly, 1,000 g of lemon flavedo tissue was distilled with 2 L of water until no more essential oil was obtained. The collected CPLEO and HDLEO were dried using anhydrous sodium sulfate and stored at −20°C. The essential oil samples were diluted in hexane at appropriate volume ratios and filtered through a 0.20 μm filter prior to HPLC analysis. For use in animal experiments, the essential oil samples were diluted in olive oil at a volume fraction of 10%.

Fifteen HPLC-grade standards of coumarins and furanocoumarins were used to identify and quantify the non-volatile constituents of the essential oils. Xanthotoxol, herniarin, oxypeucedanin hydrate, isomeranzin, auraptene, 5-geranyloxy-7-methoxy-coumarin, isopimpinellin, oxypeucedanin, imperatorin, phellopterin, 8-geranyloxy-psoralen, byakangelicol, heraclenin, and cnidicin were purchased from Yuanye Biotech (Shanghai, China). Bergaptol, citropten, and bergamottin were purchased from ChromaDex (Irvine, CA, USA). Bergapten was purchased from Toronto Research Chemicals (Toronto, ON, Canada). The internal standards (ISs), coumarin and psoralen, were procured from Yuanye Biotech (Shanghai, China). Stock solutions of ~1 mg/mL of the individual standards were prepared in 50% methanol/50% dimethyl formamide (v/v) and stored at 4°C. The purity of each reference standard was verified to be ≥98% with a QSight LX50 UHPLC and a QSight 210 MD triple quadrupole tandem mass spectrometer (PerkinElmer Inc., Waltham, MA, USA).

HPLC-grade acetonitrile, methanol and tetrahydrofuran (THF) were purchased from Honeywell (Morris Plains, NJ, USA). HPLC-grade phosphoric acid was purchased from Kelong Co. (Chengdu, Sichuan, China). Water was freshly purified using a Milli-Q Plus Advantage A10 system (Molsheim, France).

### HPLC Analysis of Coumarins and Furanocoumarins in the Lemon Essential Oils

The analytical method was adapted with minor modifications from our previous study (17). Briefly, an Agilent Poroshell EC-C8 column (i.d. 4.6 × 150 mm, 2.7 μm) was used with an Agilent 1,260 Infinity chromatography system consisting of a quaternary pump, an autosampler, a thermostatic column compartment and a photodiode array detector (PDA). The solvents consisted of a 0.05% (v/v) phosphoric acid aqueous solution (A), methanol (B), acetonitrile (C) and a 50% (v/v) aqueous solution of THF (D), and the flow rate was 1 mL/min. The solvent program consisted of the following isocratic periods: 0–7 min, 33% B, 4% C and 0% D; 917 min, 32% B, 5% C and 6% D; 20–36 min, 0% B, 34% C and 26% D; 4,146 min, 0% B, 73% C and 0% D; 5,055 min, 0% B, 100% C and 0% D; and 5,580 min, initial conditions. Solvent A composed the unlisted percentage of the eluent, and a linear gradient was set to connect each of the neighboring isocratic periods. The column temperature was maintained at 30°C. The PDA was set to scan at a wavelength of 210–400 nm with a sampling frequency of 2.5 Hz. The monitoring wavelength was set to 330 nm. The full ultraviolet (UV) absorbance spectrum of each standard compound was determined, and the spectra were compiled in a homemade library. To identify the constituents, the full UV spectra and retention times of the sample peaks were compared with those of the standards. Quantitation was carried out using an internal standard method. Coumarin and psoralen were added as the ISs for coumarins and furanocoumarins, respectively, to each dilution of the standard calibration solution at a concentration of 100 mg/L, and the correction factor (CF) was determined. The same amounts of ISs were added to the essential oils, and the sample compounds were quantitated using the CFs.

### Experimental Animal Model

Six-week-old male Kunming mice weighing ~30 ± 2 g were purchased from the experimental animal center of Chongqing Medical University of China. They were reared in a controlled room (temperature 25 ± 2°C, relative humidity 50 ± 5%, 12 h/12 h light/dark cycle). The mice were allowed access to standard food and drinking water *ad libitum*. The bedding was changed every 2 days for a week of adaptive rearing. After adaptation, the mice were randomly divided into 6 groups with 10 mice in each group. During the whole experiment, the daily food intake and body weights of the mice were recorded every day. In the first week, the mice in the normal group and D-galactose group were given 0.1 mL of normal saline by gavage; the mice in the Vc (positive control) group were treated with 0.1 mL of Vc (100 mg/kg) by gavage; the mice in the olive oil (negative control) group were treated with 0.1 mL of olive oil by gavage; the mice in the 10% CPLEO group were treated with 0.1 mL of 10% CPLEO (diluted with olive oil) by gavage; and the mice in the 10% HDLEO group were treated with 0.1 mL of 10% HDLEO (diluted with olive oil) by gavage. From the second week to the fourth week, equal volumes of D-galactose (120 mg/kg) were injected daily into the mice in all groups except the normal group (16). After the last administration, the mice were fasted for 24 h but allowed to drink freely. Blood was collected from the eyeball, and the liver was dissected for later use. These experimental schemes were approved by the ethics committee of Chongqing Collaborative Innovation Center for Functional Food, Chongqing Engineering Research Center of Functional Food. In addition, they complied with directive 2010/63/EU.

### Histopathological Analysis of Liver Tissue

After dissection, the right lobe of the liver was fixed in 10% formalin, dehydrated with 95% (v/v) ethanol, cleared with xylene, embedded in paraffin, cooled, and sectioned. The sections were fixed on slides. The embedded liver tissues were stained with hematoxylin and eosin (H&E), and the morphological changes were observed under a light microscope (BX43, Olympus, Tokyo, Japan).

### Detection of Animal Serum Indexes

Blood was centrifuged at 4,000 rpm for 10 min at 4°C, and then the serum was collected and stored at −80°C for future use. The serum total antioxidant capacity (T-AOC) and nitric oxide (NO) levels were determined by using an appropriate biochemical kit (Nanjing Jiancheng Bioengineering Institute, Nanjing, China) according to the procedures recommended by the manufacturer.

### Enzyme-Linked Immunosorbent Assay (ELISA)

Blood was centrifuged at 4,000 rpm for 10 min at 4°C, and then the serum was collected and stored at −80°C for future use. The serum levels of superoxide dismutase (SOD), glutathione (GSH), GSH peroxidase (Px), cyclooxygenase-2 (COX-2), interleukin (IL)-6, IL-1β, interferon (IFN)-γ and tumor necrosis factor (TNF)-α were determined according to the corresponding kits' instructions (Shanghai Enzyme Linked Biotechnology Co., Ltd., Shanghai, China).

### Quantitative Real-Time PCR (RT-qPCR) Assay

The expression of messenger RNA (mRNA) in mouse livers was determined by the SYBR Green method. The liver tissues of mice were crushed and then homogenized with TRIzol reagent (Thermo Fisher Scientific, Waltham, MA, USA) to extract total RNA from liver tissue. The RNA concentration was determined by a micro-UV spectrophotometer (Nano 300, Ao Sheng, Hangzhou, Zhejiang, China). Then, 2 μL of cDNA template, 10 μL of SYBR Green PCR Master Mix (Thermo Fisher Scientific), 1 μL of upstream primer and 1 μL of downstream primer were mixed, and a StepOnePlus Real-Time PCR System (Thermo Fisher Scientific, Waltham, MA, USA) was used. The PCR conditions were as follows: predenaturation at 95°C for 3 min and 40 cycles of denaturation at 95°C for 10 s, annealing at 57°C for 30 s, and extension at 72°C for 15 s. Using β-actin as a control, the relative transcription levels of mRNA were calculated by the 2^−Δ*ΔCT*^ method (18). Table 1 shows the primer sequence information for this study.

**Table 1:** Primer sequences for the RT-qPCR assay.

**Gene name**	**Sequence**
Cu/Zn SOD	Forward: 5′-AACCAGTTGTGTTGTCAGGAC-3′
	Reverse: 5′-CCACCATGTTTCTTAGAGTGAGG-3′
Mn SOD	Forward: 5′-AGACCTGCCTTACGACTATGG-3′
	Reverse: 5′-CTCGGTGGCGTTGAGATTGTT-3′
CAT	Forward: 5′-TGGCACACTTTGACAGAGAGC-3′
	Reverse: 5′-CCTTTGCCTTGGAGTATCTGG-3′
HO-1	Forward: 5′-GATAGAGCGCAACAAGCAGAA-3′
	Reverse: 5′-CAGTGAGGCCCATACCAGAAG-3′
Nrf2	Forward: 5′-TAGATGACCATGAGTCGCTTGC-3′
	Reverse: 5′-GCCAAACTTGCTCCATGTCC-3′
NQO1	Forward: 5′-AGGATGGGAGGTACTCGAATC-3′
	Reverse: 5′-TGCTAGAGATGACTCGGAAGG-3′
nNOS	Forward: 5′-TCCCAGTAACGGACCTCAG-3′
	Reverse: 5′-TGCTCAACACAGGTTCTATCTC-3′
iNOS	Forward: 5′-GGAGTGACGGCAAACATGACT-3′
	Reverse: 5′-TCGATGCACAACTGGGTGAAC-3′
IL-1β	Forward: 5′-GAAATGCCACCTTTTGACAGTG-3′
	Reverse: 5′-TGGATGCTCTCATCAGGACAG-3′
COX-2	Forward: 5′-TGCACTATGGTTACAAAAGCTGG-3′
	Reverse: 5′-TCAGGAAGCTCCTTATTTCCCTT-3′
NF-κB	Forward: 5′-GGGGCCTGCAAAGGTTATC-3
	Reverse: 5′-TGCTGTTACGGTGCATACCC-3
TNF-α	Forward: 5′-GAGGCCAAGCCCTGGTATG-3′
	Reverse: 5′-CGGGCCGATTGATCTCAGC-3′
β-actin	Forward: 5′-GCCGACAGGATGCAGAAGG-3′
	Reverse: 5′-TGGAAGGTGGACAGCGAGG-3′

*Cu/Zn-SOD, copper/zinc superoxide dismutase; Mn-SOD, manganese superoxide dismutase; CAT, catalase; HO-1, heme oxygenase-1; Nrf2, nuclear factor E2-related factor 2; NQO1, NAD(P)H quinone dehydrogenase 1; nNOS, neuronal nitric oxide synthase; iNOS, inducible nitric oxide synthase; IL-1β, interleukin-1 beta; COX-2, cyclooxygenase-2; NF-κB, nuclear factor kappa-B; TNF-α, tumor necrosis factor alpha*.

### Western Blot Analysis

A total of 100 mg of liver tissue sample was collected and homogenized with 1 mL of radioimmunoprecipitation assay (RIPA) buffer and 10 μL of phenylmethylsulfonyl fluoride (PMSF). The sample was then centrifuged at 4°C at 12,000 rpm for 4 min. The middle protein layer was collected, and the protein was quantified with a bicinchoninic acid (BCA) kit. Each sample was diluted to 40 μg/mL; then, the diluted protein was mixed with sample buffer, and the mixture was heated at 95°C for 10 min for denaturation. A prestained protein ladder and the samples were loaded into the sample wells of an SDS-polyacrylamide gel and subjected to vertical SDS-PAGE. The proteins were then transferred to a polyvinylidene fluoride (PVDF) membrane. Then, the PVDF membrane was sealed with 5% skim milk in Tris buffer containing Tween 20 (TBST) solution for 1 h. After sealing, the PVDF membrane was washed with TBST and incubated with primary antibodies against copper/zinc SOD (Cu/Zn-SOD), manganese SOD (Mn-SOD), catalase (CAT), GSH-Px, heme oxygenase-1 (HO-1), nuclear factor E2-related factor 2 (Nrf-2), COX-2, NO synthase (NOS) 1, NOS2, NOS3, and β-actin (Santa Cruz Biotechnology Inc., Santa Cruz, CA, USA) at 4°C overnight. After that, the PVDF membrane was washed with TBST and then incubated with the corresponding secondary antibodies at 25°C for 1 h. According to the instructions of a SuperSignal West Pico Plus Kit, a chromogenic solution was prepared and used to completely cover the PVDF membrane; the membrane was then put into an iBright FL1000 imaging system (Thermo Fisher Scientific) for observation (18). In addition, ImageJ software was used to analyze the images, and β-actin was used as an internal reference protein to calculate the relative expression of the target proteins.

### Statistical Analysis

Three or more parallel experiments were carried out to determine the serum and tissue indexes for each mouse, and then the average value was taken. The data were analyzed with SPSS 22 (SPSS Inc., IL, USA). The experimental results are expressed as the mean ± standard deviation (SD). The differences in mean values among groups were evaluated by one-way ANOVA using Duncan's multiple range test (MRT). A difference of *P* < 0.05 was considered to indicate statistical significance.

## Results

### Non-volatile Constituents of CPLEO and HDLEO

**Figure 1:**
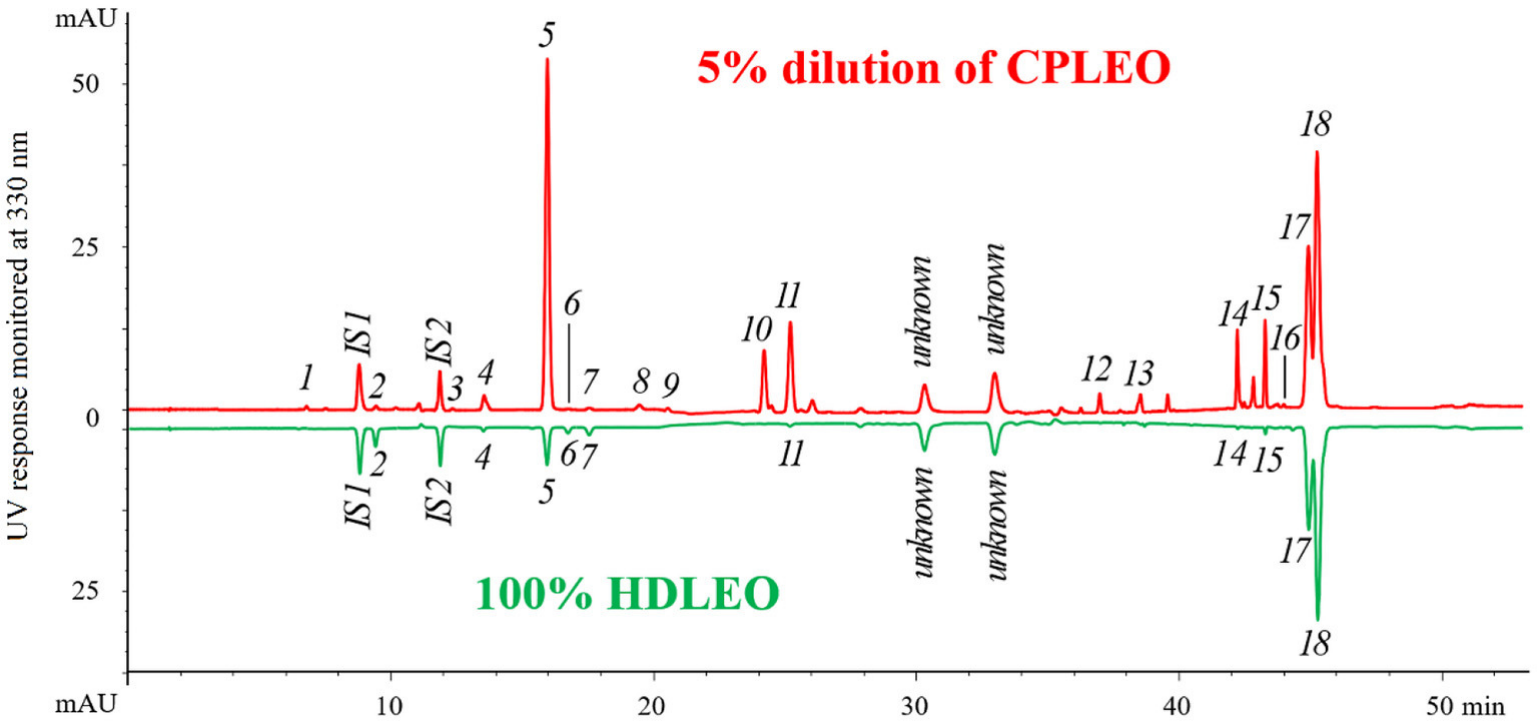
High-performance liquid chromatography (HPLC) chromatograms of cold-pressed and hydrodistilled lemon essential oils. The peak assignment corresponds to that in Table 1. IS1 (coumarin) and IS2 (psoralen) are internal standards.

The results of separation and identification of the non-volatile components in both lemon essential oils are shown in Figure 1. The UV chromatogram of a 5% (v/v) CPLEO dilution is shown in the upper half, and that of pure HDLEO, inverted for better comparison, is shown in the lower half. Most of the major peaks in the oil samples were identified. Two unknown peaks at ~30 and 33 min in both samples were determined to not be coumarin or furanocoumarin *via* assessment of their UV spectra. All the 18 target compounds were found in CPLEO. Except for compounds *17* and *18*, the other 16 compounds and the added ISs were separated at baseline. By carefully searching references (18), we found that xanthotoxol and isomeranzin were identified for the first time in the cold-pressed essential oil of Eureka lemon. Ten compounds were detected in HDLEO. They were coevaporated with the volatiles during distillation.

The results of quantitation are shown in Table 2. Generally, the few coumarins and furanocoumarins found in HDLEO had very low concentrations, and their total content was in the tens of ppm range. The total amount of target components in CPLEO was more than 200 times higher than those in HDLEO. Among the CPLEO components, the most abundant compounds were 8-geranyloxypsoralen and bergamottin, followed by 5-geranyloxy-7-methoxycoumarin, citropten, oxypeucedanin and byakangelicol; the concentrations of these compounds were all higher than 1.0 g/L. Furanocoumarins accounted for ~2/3 of the total mass, while coumarins accounted for the remaining 1/3.

**Table 2:** Quantitation of 18 coumarins and furanocoumarins in cold-pressed and hydrodistilled lemon essential oils.

**No**.	**Rt (min)**	**Type**	**Compound name**	**Index of quantitation**					**Concentration**	
				**Linear Range (mg/L)**	**Correlation (*R*^**2**^)**	**CF**	**LOD (mg/L)**	**LOQ (mg/L)**	**CPLEO (mg/L)**	**HDLEO (mg/L)**
1	6.8	FC	xanthotoxol	2.0–1500	0.9998	7.30	0.68	2.27	24.2 ± 0.4	ND
2	9.4	C	herniarin	0.5–600	0.9996	7.75	0.31	1.05	22.1 ± 1.3	1.32 ± 0.01
3	12.3	FC	bergaptol	0.1–500	0.9997	23.3	0.02	0.08	18.1 ± 2.2	ND
4	16.5	FC	oxypeucedanin hydrate	0.5–500	0.9999	6.14	0.16	0.52	9.9 ± 0.6	0.97 ± 0.01
5	15.9	C	citropten	0.1–500	1.0000	12.2	0.02	0.06	1794.3 ± 3.2	1.93 ± 0.01
6	16.6	FC	isopimpinellin	0.5–150	0.9999	4.09	0.03	1.00	16.9 ± 0.5	1.47 ± 0.01
7	17.5	FC	bergapten	0.3–500	0.9994	19.8	0.03	1.00	27.5 ± 0.7	1.25 ± 0.00
8	19.4	FC	heraclenin	0.5–1,200	1.0000	16.3	0.22	0.73	208.5 ± 0.8	ND
9	20.8	C	isomeranzin	0.05–500	0.9996	8.47	0.04	0.14	3.9 ± 0.6	ND
10	24.2	FC	byakangelicol	1.0–4,000	1.0000	4.15	0.20	0.67	1392.6 ± 3.3	ND
11	25.2	FC	oxypeucedanin	1.0–500	0.9995	24.5	0.04	0.14	1415.2 ± 0.4	0.43 ± 0.04
12	37.0	FC	imperatorin	0.2–200	0.9998	13.5	0.02	0.05	265.9 ± 7.6	ND
13	38.5	FC	phellopterin	0.2–200	0.9996	4.58	0.01	0.04	86.1 ± 4.4	ND
14	42.2	FC	cnidicin	0.2–300	0.9999	5.37	0.02	0.07	512.4 ± 2.1	20.7 ± 0.1
15	43.3	FC	8-geranyloxy-psoralen	0.2–200	0.9999	10.2	0.01	0.04	3348.2 ± 14.7	2.96 ± 0.00
16	44.0	C	auraptene	1.0–500	0.9999	4.54	0.03	0.11	23.3 ± 0.4	ND
17	44.9	FC	bergamottin	0.5–500	0.9997	22.4	0.02	0.06	3268.7 ± 13.5	15.1 ± 0.4
18	45.3	C	5-geranyloxy-7-methoxycoumarin	0.5–500	1.0000	5.46	0.12	0.40	2312.1 ± 9.6	18.1 ± 0.0
Total									14.7 g/L	64.2 mg/L

*ND, not detected; C, coumarins; FC, furanocoumarins*.

### Histopathological Evaluation of Liver Tissues

**Figure 2:**
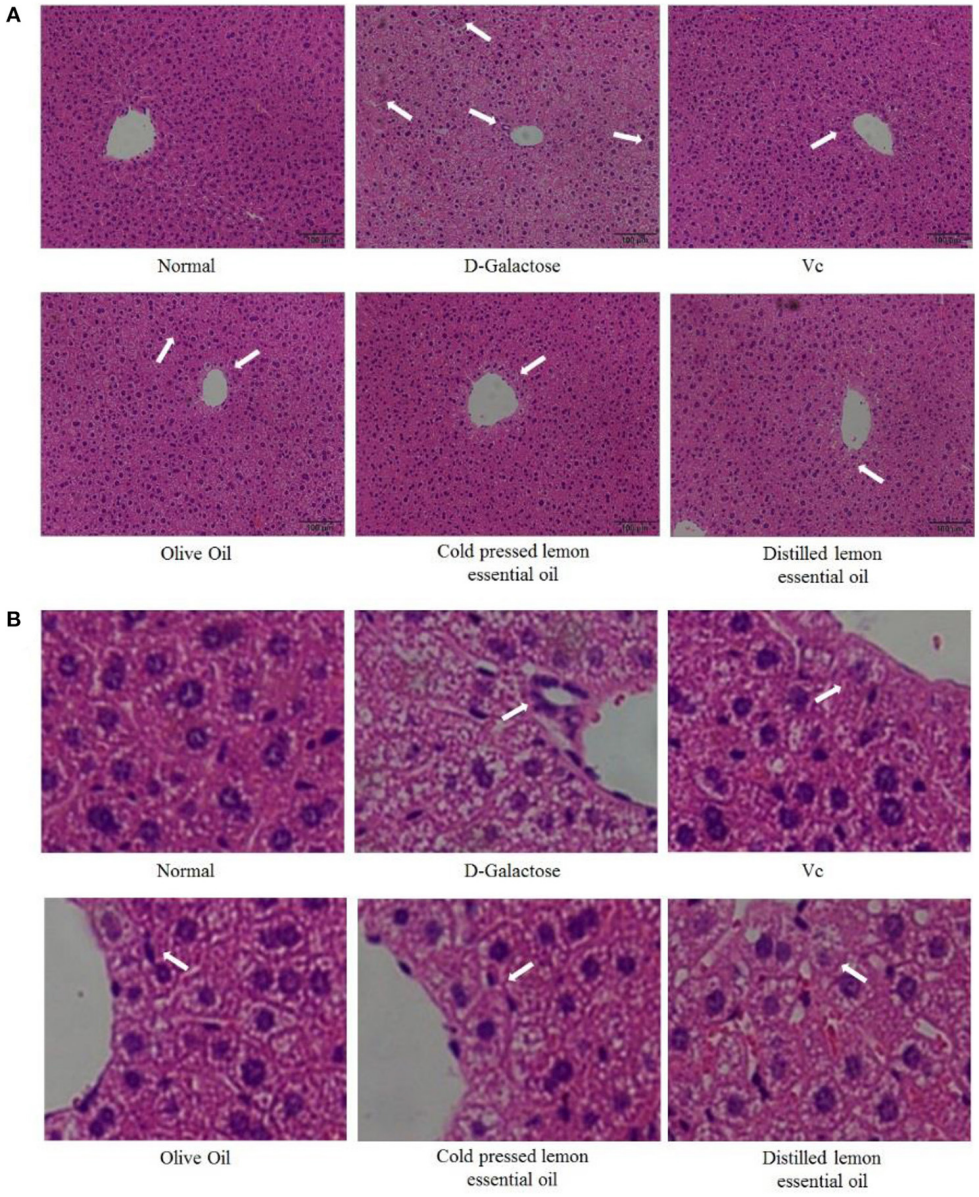
Pathological observation of hematoxylin-eosin (H&E)-stained sections of liver tissue from mice with oxidative damage. The area indicated by the arrow is inflammatory infiltration. **(A)** 100 × ,**(B)** 600 × .

Figure 2 shows H&E-stained sections of mouse liver. Under normal conditions, the morphology and structure of hepatocytes in mouse liver sections were normal and complete, and the cells were linearly distributed around the central vein. There was no inflammatory cell infiltration, and the hepatic sinuses were not dilated or congested. However, in the D-galactose group, hepatocytes were disordered, irregular in shape, and lacked cell boundaries, and swelling and extensive signs of inflammatory infiltration were visible. Compared with those in the D-galactose group, the hepatocytes in the olive oil group did not exhibit significantly improved morphology. The hepatocytes in CPLEO-, HDLEO-, and Vc-treated mice were relatively intact, with orderly arrangement of hepatocytes, intact hepatic cords and reduced inflammatory cell infiltration. Moreover, the hepatocytes in the CPLEO group showed clear nuclear structures with only small amounts of cell infiltration, similar to those in the positive control (Vc) group and to the normal hepatocyte morphology, indicating that CPLEO prevented the damage to liver tissue caused by D-galactose oxidation more effectively than HDLEO.

### Levels of the Oxidative Indicators T-AOC, SOD, GSH, and GSH-Px in Mouse Serum

**Figure 3:**
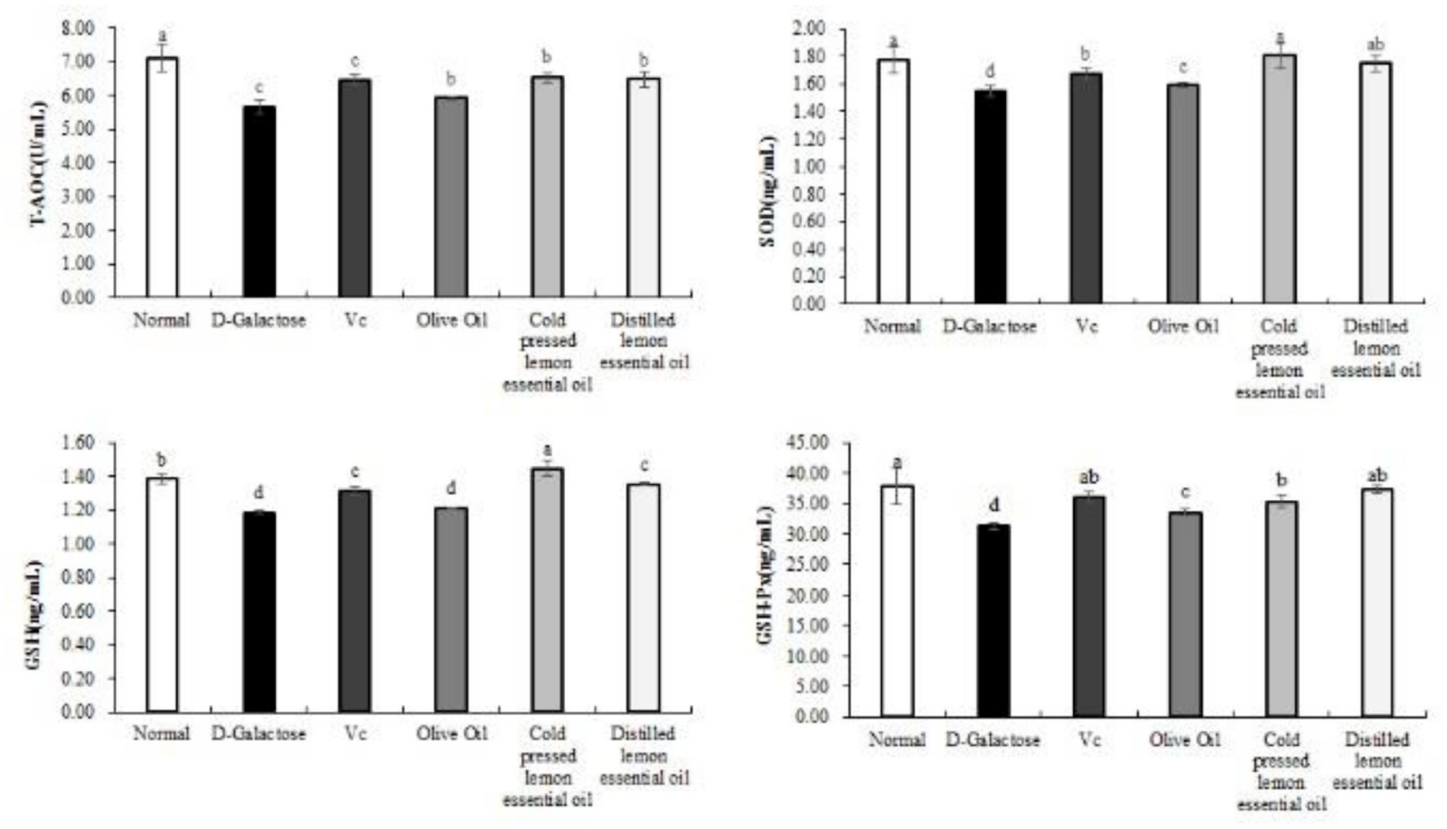
Total antioxidant capacity (T-AOC), superoxide dismutase (SOD), glutathione (GSH) and glutathione peroxidase (GSH-Px) levels in the serum of mice with oxidative damage. ^a−*d*^Mean values with different letters in the same bar are significantly different (*p* < 0.05) according to Duncan's multiple range test.

The levels of T-AOC, SOD, GSH, and GSH-Px in the serum of mice are shown in Figure 3. The levels of all indicators were highest in the normal group and lowest in the D-galactose group. In addition, the serum levels of T-AOC, SOD, GSH and GSH-Px in the Vc, CPLEO and HDLEO groups were significantly (*P* < 0.05) higher than those in the D-galactose and olive oil groups. Furthermore, the serum T-AOC, SOD and GSH levels in the CPLEO group were relatively similar to those in the normal group and were significantly higher than those in the Vc group (*P* < 0.05). These results suggest that CPLEO can protect against oxidative stress induced by D-galactose by regulating oxidative markers in serum.

### Serum Levels of the Inflammatory Indicators NO, COX-2, IL-6, IL-1β, IFN-γ, and TNF-α in Mice

**Figure 4:**
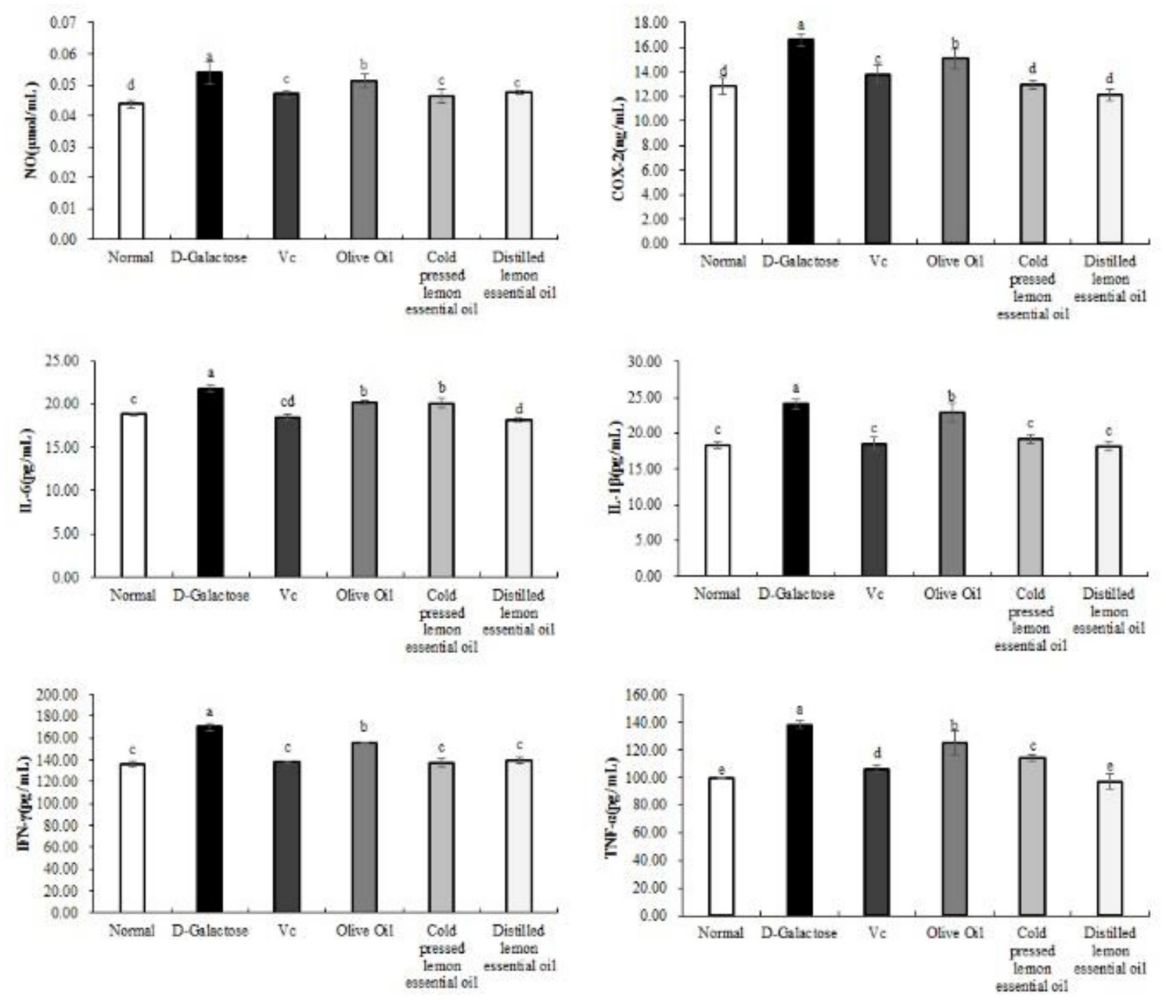
Nitric oxide (NO), cyclooxygenase-2 (COX-2), interleukin-6 (IL-6), interleukin-1 beta (IL-1β), interferon gamma (IFN-γ) and tumor necrosis factor alpha (TNF-α) levels in the serum of mice with oxidative damage. ^a−*e*^Mean values with different letters in the same bar are significantly different (*p* < 0.05) according to Duncan's multiple range test.

Figure 4 shows the expression results for the inflammatory indicators NO, COX-2, IL-6, IL-1β, IFN-γ, and TNF-α in mouse serum. The mice in the normal group had the lowest serum levels of all inflammatory indexes, while those in the D-galactose group had the highest levels. Compared with the D-galactose group, the Vc, olive oil, CPLEO, and HDLEO oil groups showed significant (*P* < 0.05) improvements in serum inflammatory indicators, and the serum levels of inflammatory indicators in the CPLEO and HDLEO groups were relatively similar to those in the normal group and were lower than those in the VC group. This result suggests that lemon essential oil may alleviate the aging effect caused by D-galactose by suppressing inflammatory responses.

### mRNA Expression of the Oxidation-Related Genes in Mouse Liver Tissues

**Figure 5:**
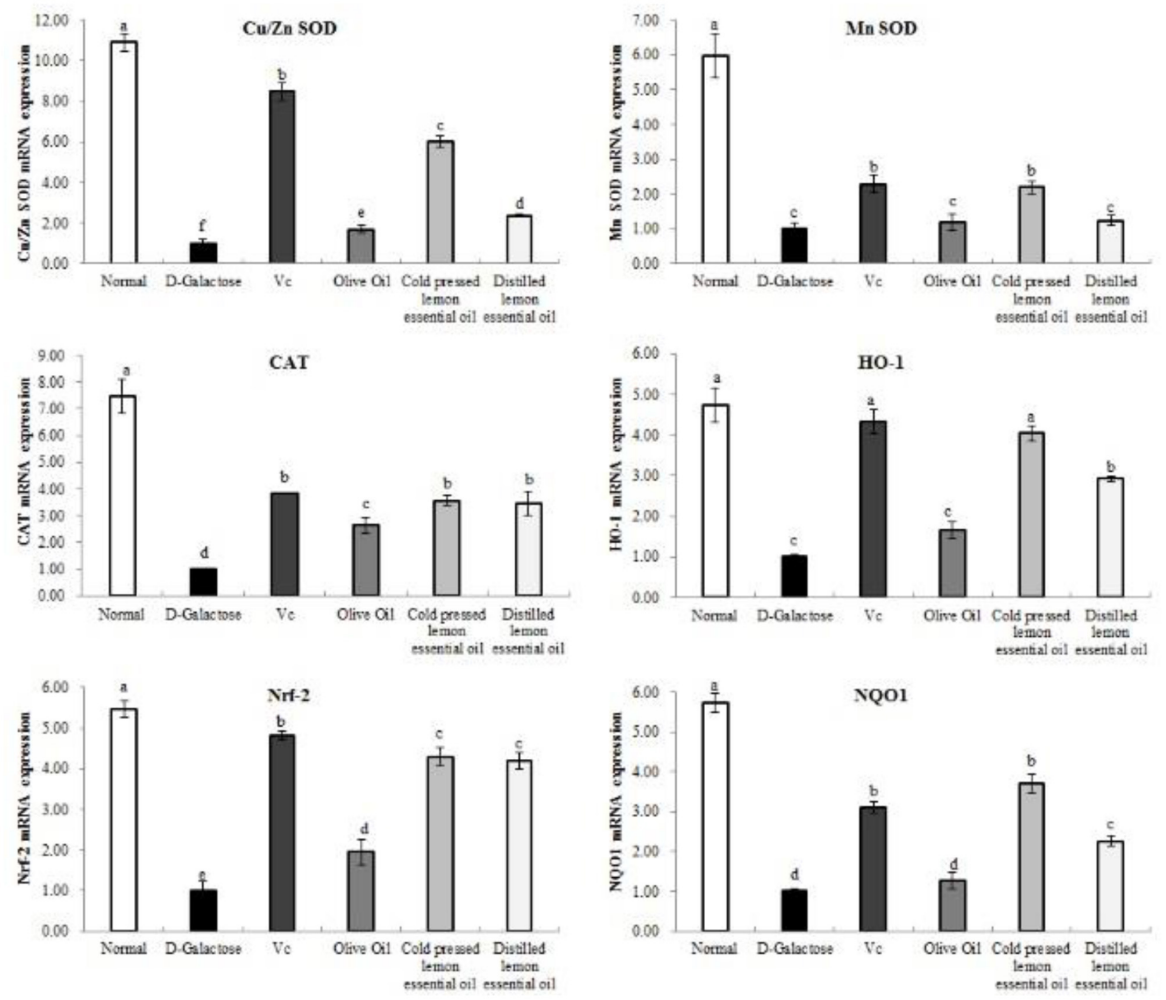
Copper/zinc superoxide dismutase (Cu/Zn SOD), manganese superoxide dismutase (Mn SOD), catalase (CAT), heme oxygenase-1 (HO-1), nuclear factor E2-related factor 2 (Nrf2) and NAD(P)H quinone dehydrogenase 1 (NQO1) mRNA expression in the liver tissues of mice with oxidative damage. ^a−*f*^Mean values with different letters in the same bar are significantly different (*p* < 0.05) according to Duncan's multiple range test.

**Figure 6:**
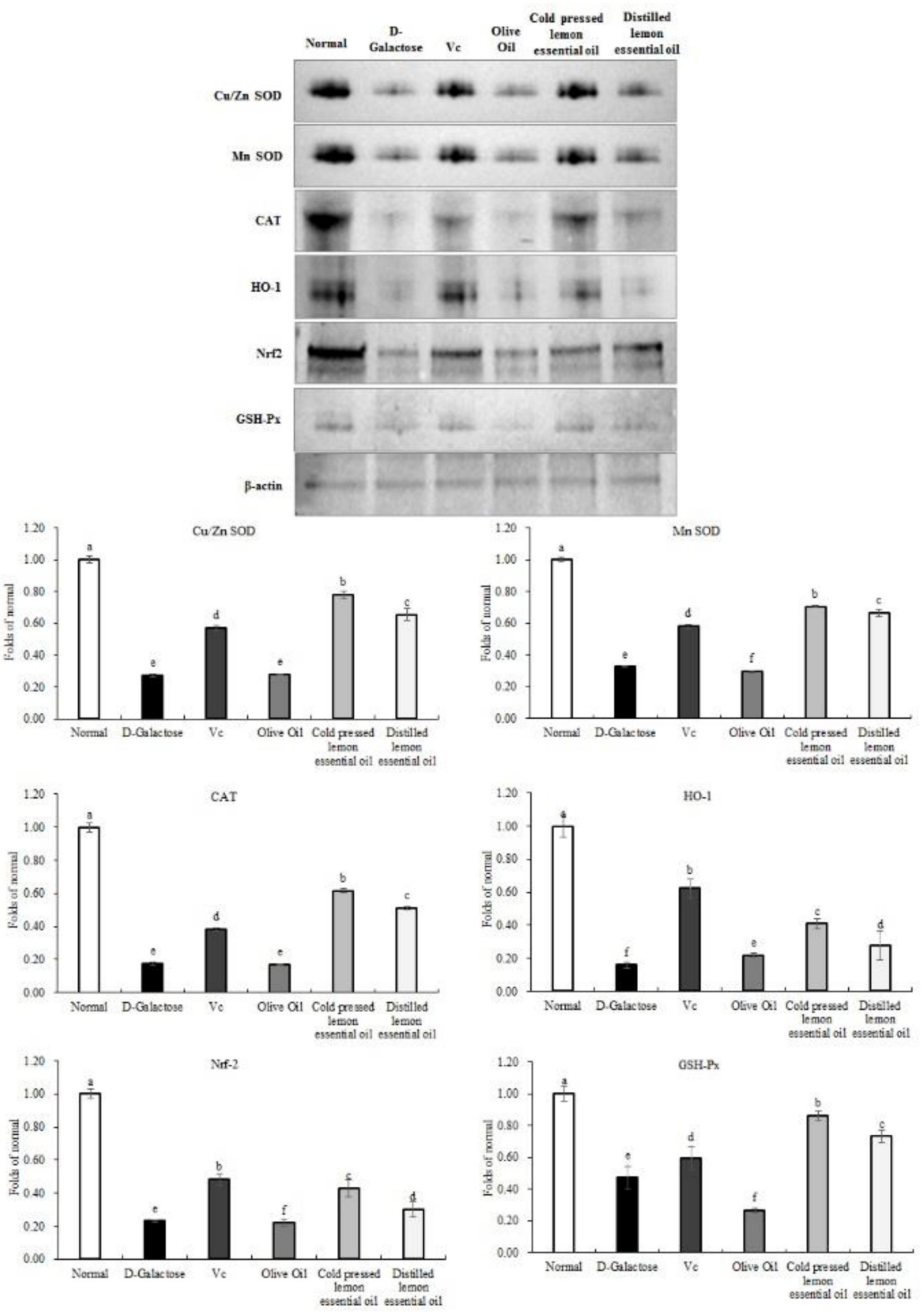
Copper/zinc superoxide dismutase (Cu/Zn SOD), manganese superoxide dismutase (Mn SOD), catalase (CAT), heme oxygenase-1 (HO-1), nuclear factor E2-related factor 2 (Nrf2) and NAD(P)H quinone dehydrogenase 1 (NQO1) protein expression in the liver tissues of mice with oxidative damage. ^a−*f*^Mean values with different letters in the same bar are significantly different (*p* < 0.05) according to Duncan's multiple range test. β-actin is the internal reference expression. As the same experiment, the β-actin internal parameters of this Figure 6 and the β-actin internal parameters of **Figure 8** is the same.

As shown in Figures 5, 6, the normal group, Vc group, and CPLEO group all showed high mRNA expression of Cu/Zn-SOD, Mn-SOD, CAT, HO-1, Nrf-2, and NQO1 in liver tissue and high protein expression of Cu/Zn-SOD, Mn-SOD, CAT, GSH-Px, HO-1, and Nrf-2. Among the groups, the normal group showed the highest expression of oxidation indicators, and the Vc, CPLEO, and HDLEO groups showed significantly (*P* < 0.05) higher expression than the D-galactose and olive oil groups. Moreover, the liver mRNA expression of Cu/Zn-SOD, Mn-SOD, HO-1, and NQO1 and the liver protein expression of Cu/Zn-SOD, Mn-SOD, CAT, HO-1, Nrf-2, and GSH-Px were significantly (*P* < 0.05) higher in the CPLEO group than in the HDLEO group. Cu/Zn-SOD, Mn-SOD, CAT, and GSH-Px protein expression and NQO1 mRNA expression were even significantly (*P* < 0.05) higher in the CPLEO group than in the Vc group. These data suggest that CPLEO can effectively regulate the expression of Cu/Zn-SOD, Mn-SOD, CAT, HO-1, Nrf-2, NQO1, and GSH-Px in organisms; reduce free radical damage caused by D-galactose and lipid peroxidation; and balance the oxidative stress response in the body.

### mRNA Expression of the Inflammation-Related Genes in Mouse Liver Tissues

**Figure 7:**
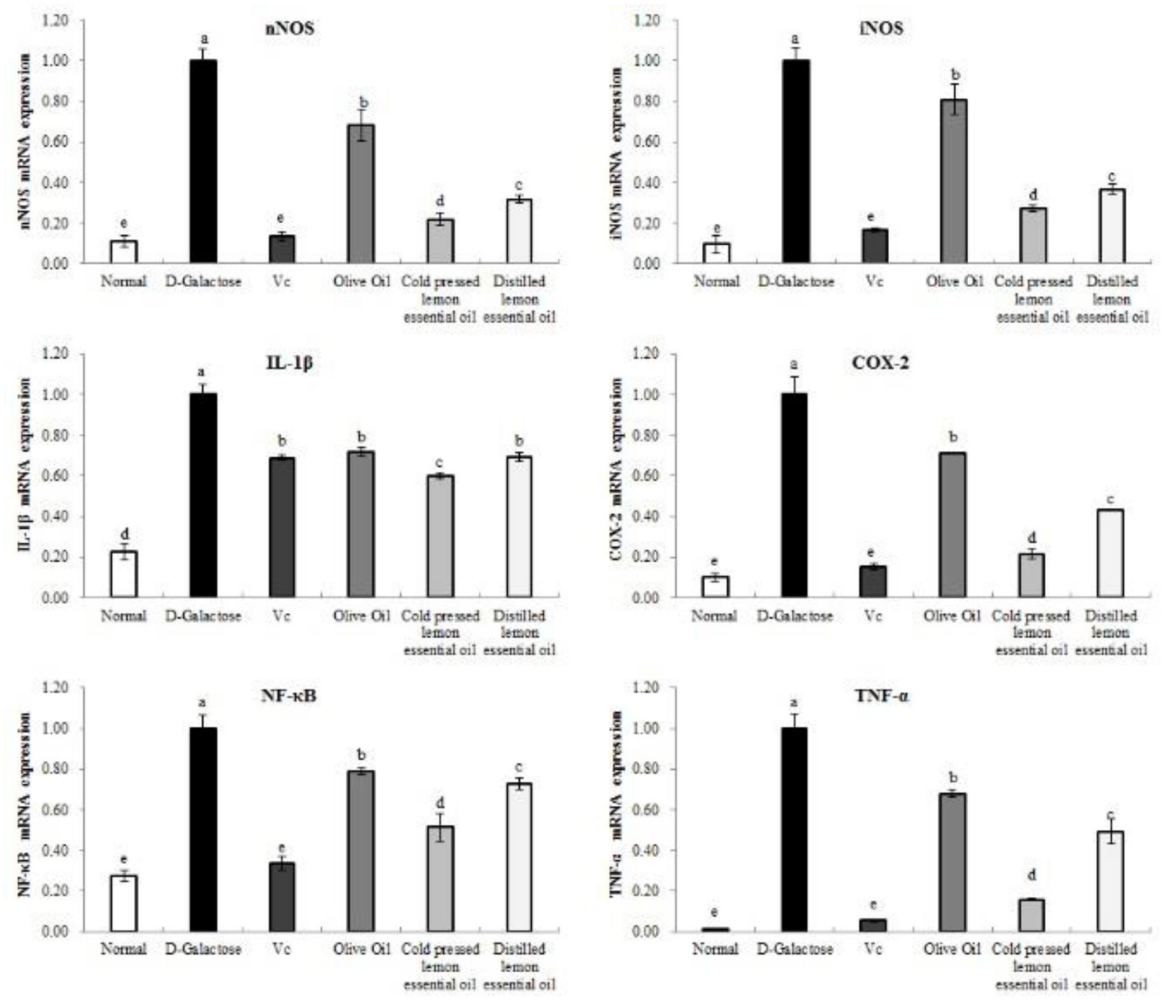
Neuronal nitric oxide synthase (nNOS), inducible nitric oxide synthase (iNOS), interleukin-1 beta (IL-1β), cyclooxygenase-2 (COX-2), tumor necrosis factor alpha (TNF-α), and nuclear factor kappa-B (NF-κB) mRNA expression in the liver tissues of mice with oxidative damage. ^a−*e*^Mean values with different letters in the same bar are significantly different (*p* < 0.05) according to Duncan's multiple range test.

**Figure 8:**
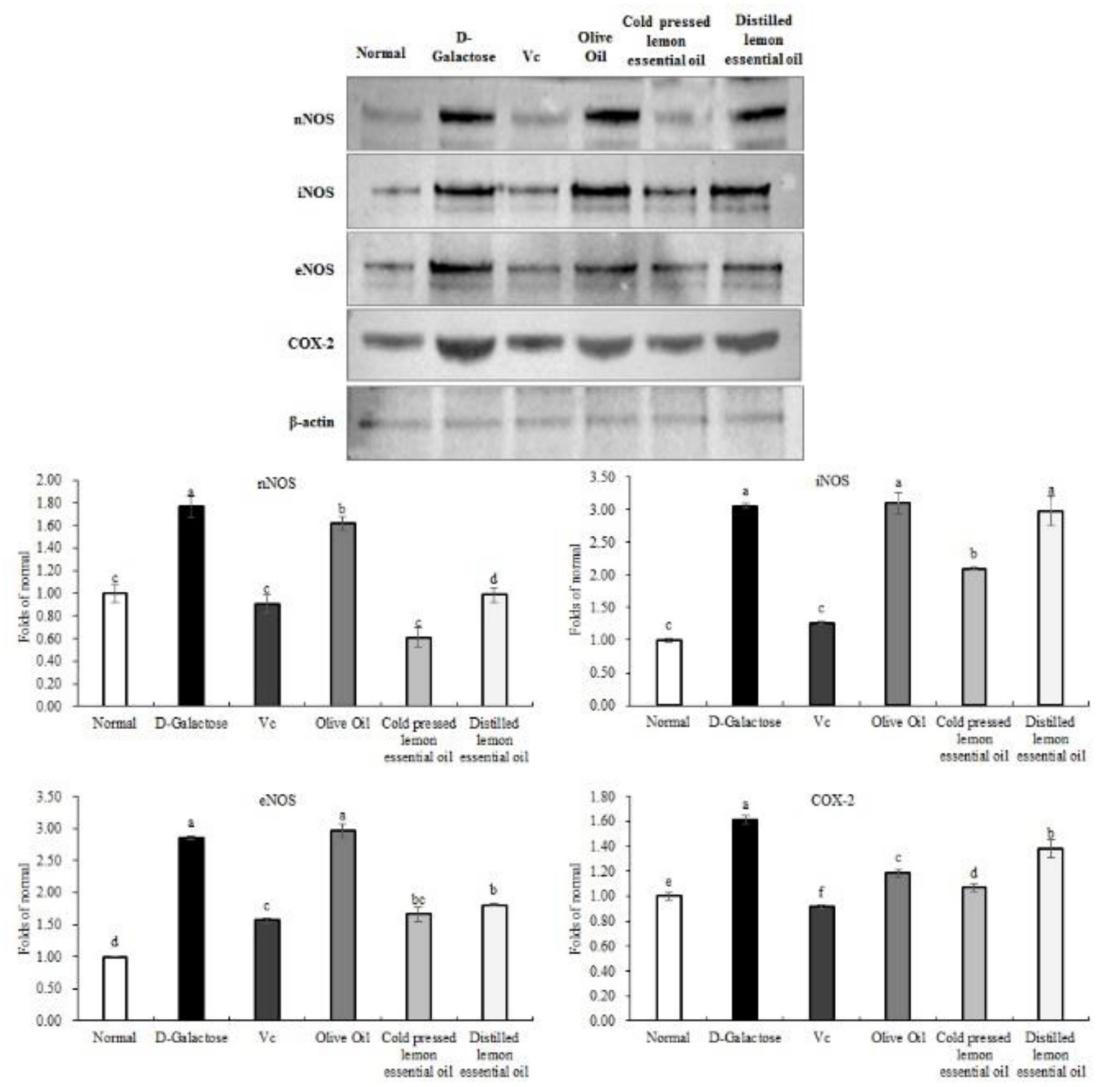
Neuronal nitric oxide synthase (nNOS), endothelial nitric oxide synthase (eNOS), inducible nitric oxide synthase (iNOS), and cyclooxygenase-2 (COX-2) protein expression in the liver tissues of mice with oxidative damage. ^a−*f*^Mean values with different letters in the same bar are significantly different (*p* < 0.05) according to Duncan's multiple range test. β-actin is the internal reference expression. As the same experiment, the β-actin internal parameters of Figure 6 and the β-actin internal parameters of this figure is the same.

As shown in Figures 7, 8, the mRNA expression levels of nNOS, iNOS, IL-1β, COX-2, NF-κB, and TNF-α and the protein expression levels of iNOS, eNOS, and COX-2 in mouse liver tissues were lowest in the normal group and highest in the D-galactose group. The liver IL-1β mRNA expression in the CPLEO group was closest to that in the normal group and was significantly (*P* < 0.05) lower than that in the Vc group. The liver mRNA expression of nNOS, iNOS, COX-2, NF-κB, and TNF-α in the Vc group was the closest to that in the normal group. In addition, the expression in the CPLEO group was only higher than that in the Vc group and was significantly (*P* < 0.05) lower than that in the HDLEO group. Similar results were obtained for the protein expression of iNOS and eNOS, which were only higher in the CPLEO group than in the Vc group and were significantly (*P* < 0.05) lower in the CPLEO group than in the HDLEO group. Moreover, the protein expression of nNOS in the CPLEO group was even lower than that in the normal group (*P* > 0.05), and the protein expression of COX-2 was only higher than that in the normal group. The above data suggest that CPLEO can effectively balance the inflammatory response to alleviate aging induced by D-galactose.

## Discussion

Free radicals are the intermediate products of normal metabolism. They have strong reactivity and can oxidize many substances in cells and damage biofilms (19). Oxidative stress, which is the result of increased production and/or decreased scavenging ability of free radicals, impairs the oxidative and antioxidant systems in organisms, leading to oxidative damage *via* accumulation of free radicals and this process is also an important aspect of aging (20). Plant essential oils have been confirmed to have good antioxidant effects, and phenolic components in plant essential oils are highly reactive with peroxyl radicals; specifically, they donate reactive hydrogen atoms or electrons to free radicals, turning free radicals into less active species and clearing them (21). These effects are attributable to the redox characteristics and chemical structures of phenolics in the essential oils (22). For example, the transition metals Fe, Cu, etc. are catalysts of many free radical-generating processes, and certain essential oil phenolics can combine with these transition metals to prevent metal-induced free radical generation (23). In addition, some active antioxidant substances in plant essential oils can bind to receptors on cells and increase the secretion of antioxidant enzymes through signal transduction, thus enhancing antioxidant defense capacity (24). Overall, the effects of plant essential oils on free radicals and damaged tissues result in inhibition or delay of aging.

SOD, T-AOC, GSH, and GSH-Px are key indicators of oxidation (25, 26). The levels of oxidative stress can be determined through analysis of human or animal serum. T-AOC can be used to estimate antioxidant levels in serum, and SOD and GSH-Px are important antioxidant enzymes; GSH is also an important antioxidant that can remove free radicals (26, 27). Both CPLEO and HDLEO significantly increased the levels of these antioxidant indexes in the serum of mice with oxidative damage, but CPLEO had the best effect; it was better even than Vc.

After the occurrence of oxidative stress, imbalance between oxidation and antioxidation leads to inflammatory infiltration of central granulocytes and to production of numerous proinflammatory factors, including IL-6, IL-1β, IFN-γ, TNF-α, and NF-κB. Therefore, the systemic levels of these factors can reflect the degree of oxidative imbalance (28). COX-2 is the key link that triggers the inflammatory response. After inflammation is aggravated by oxidative damage, the levels of COX-2 also increase significantly (29). Increases in NO levels can promote production of iNOS and increases in COX-2 (30). Therefore, the levels of COX-2 and NO can be reduced to control inflammation, thus reducing oxidative stress injury and exerting antioxidant effects. Both CPLEO and HDLEO had the ability to reduce inflammation, similar to Vc.

Reactive oxygen species (ROS) are produced in the process of aerobic metabolism. Under normal physiological conditions, due to the existence of various antioxidant enzymes (GSH-Px, SOD, etc.) and non-enzymatic antioxidant substances (GSH, Vc, vitamin E, etc.), the production and elimination of ROS are in a dynamic balance (31). The content of ROS and/or RNS increased after the imbalance of oxidative stress. At the same time, the body's antioxidant defense ability decreased. Therefore, oxidative stress may be the result of the imbalance of ROS/RNS, and the above antioxidants also have a certain inhibitory effect on the imbalance of ROS/RNS (32). Under conditions of oxidative stress, electrophilic substances induce Kelch-like ECH-associated protein-1 (Keap1) to change its conformation and release Nrf2. Nrf2 is activated through the PKC pathway, MAPK pathway, PI3K pathway and other regulatory signaling pathways, and transcription of antioxidant stress protein-encoding genes then begins to protect the body from oxidative damage (33). AP-1 is a redox-sensitive protein that can activate the transcription of many genes, including HO-1, and participate in the protective response to oxidative stress (34). One study on the upstream regulatory region of the HO-1 gene promoter identified multiple antioxidant response elements (AREs) in the region adjacent to AP-1 and NF-?β, and Nrf2 is the main nuclear transcription regulator that directly binds to AREs (35). Under normal conditions, Nrf2 binds to Keap1 in the cytoplasm and is in an inactive state. Under oxidative stress, ROS phosphorylate Nrf2 and dissociate Keap1 *via* the MAPK signaling pathway, resulting in reduced ubiquitination of Nrf2, which increases Nrf2 nuclear translocation and entry. Nrf2 then forms a heterodimer with the Maf protein and binds with AREs to start the transcription of the HO-1 gene (36).

By regulating the HO-1/Nrf2 pathway, the binding of Nrf2 to AREs also induces the expression of series of downstream antioxidant enzymes, such as SOD (Cu/Zn SOD and Mn SOD), CAT and NQO1. These cytoprotective proteins are called “ultimate antioxidants” and can protect the body from oxidative-toxic substances (37–40). Lemon essential oil enhanced the mRNA and protein expression of Cu/Zn SOD, Mn SOD, CAT, and NQO1, and the effect of CPLEO was better than that of HDLEO, which was close to that of Vc.

nNOS, eNOS, and iNOS are three isozymes of NOS. NOS uses *L*-arginine and molecular oxygen as substrates and the NADPH coenzyme as a cofactor and generates NO through a series of oxidation reactions. Excessive *in vivo* NO leads to cytotoxic oxidant production, which in turn leads to oxidative stress diseases (41). It has also been shown that under oxidative stress conditions, the expression of nNOS, eNOS and iNOS is elevated, as is the expression of HO-1, which is closely related to that of NOS (42). In this study, lemon essential oil was very effective in downregulating the expression of NOS. The mechanism might have involved regulation of the NO-1/Nrf2 pathway, and the excellent effect of CPLEO, as shown in Figures 7, 8, was likely due to the abundant non-volatile active components.

We found that the non-volatile components of CPLEO included mainly 18 coumarins and furanocoumarins, whereas there were only 10 compounds in HDLEO. Because of the non-volatile nature of these compounds, little of these active components could be distilled and collected in HDLEO. Citropten, byakangelicol, oxypeucedanin, 8-geranyloxy-psoralen, bergamottin, and 5-geranyloxy-7-methoxycoumarin were the most abundant components in CPLEO, and their total molar concentrations were ~44.6 mmol/L. Citropten was shown to inhibit *in vitro* superoxide and nitric oxide generation (43). Byakangelicol can inhibit the activity and induction of COX-2 in human pulmonary epithelial cells (44). Citropten and byakangelicol are likely to contribute to the better anti-inflammatory effect of CPLEO in this study. In addition, oxypeucedanin was shown to upregulate differentially expressed genes for regulatory proteins involved in the MAPK signaling pathway in mouse neuroblastoma cells (45). Our previous study of pomelo essential oil showed a good *in vitro* scavenging ability of RNS and ROS by pomelo coumarins and furanocoumarins; we proposed the epoxy moiety and allylic hydrogen atom close to an oxygen atom were responsible for the radical scavenging effects (46, 47). Among the most abundant compounds quantified in this study, byakangelicol and oxypeucedanin possess the side chain epoxy groups, and 8-geranyloxy-psoralen, bergamottin, and 5-geranyloxy-7-methoxycoumarin all possess the allylic hydrogen atoms. Therefore, they are promising candidates for further evaluation of their effects on oxidative stress.

The antioxidant activity of lemon essential oil has long been addressed to the volatile compounds, while there has been little research on non-volatile compounds. In this study, both CPLEO and HDLEO had strong antioxidant activity, but CPLEO was significantly more effective than HDLEO to modulate the expression of most selected oxidation and inflammation-related genes and proteins, indicating that non-volatile compounds, even at a comparatively low level (mmol/L), play important roles in antioxidation. In the future, we can further screen the specific non-volatile substances or remove certain furanocoumarin which elicits side effects, to improve the efficacy and safety of CPLEO. The mechanisms by which the active components in lemon essential oil regulate the NO-1/Nrf2 pathway also need to be further studied and confirmed.

## Conclusions

In this study, CPLEO and HDLEO were compared. The cold-pressing process extracted more active non-volatile substances than hydrodistillation. Animal experiments demonstrated that the increased abundance of non-volatile substances in the CPLEO enhanced the antioxidative effectiveness of the oil. The use of cold-pressing as the extraction process for lemon essential oil thus has advantages with regard to retention of the active components of lemon peels and production of functional foods. Animal experiments showed that CPLEO effectively regulated the HO-1/Nrf2 pathway, which plays an important antioxidant role, and intervened in the oxidative damage in mice caused by D-galactose. The protective effect of CPLEO was better than that of HDLEO and similar to that of vitamin C. Lemon essential oil is an effective antioxidant and could be used as a functional food or functional food additive.

## Data Availability Statement

The original contributions presented in the study are included in the article/supplementary material, further inquiries can be directed to the corresponding author/s.

## Ethics Statement

The animal study was reviewed and approved by ethics committee of Chongqing Collaborative Innovation Center for Functional Food, Chongqing Engineering Research Center of Functional Food. In addition, they complied with directive 2010/63/EU.

## Author Contributions

GL: conceptualization, methodology, and writing—original draft. SX: writing—original draft. YP, XL, and YC: data analysis. LH and XZ: writing—review and editing. All authors contributed to the article and approved the submitted version.

## Conflict of Interest

The authors declare that the research was conducted in the absence of any commercial or financial relationships that could be construed as a potential conflict of interest.

## Abbreviations

HPLC: high-performance liquid chromatography
T-AOC: total antioxidant capacity
SOD: superoxide dismutase
GSH: glutathione
GSH-Px: glutathione peroxidase
NO: nitric oxide
COX-2: cyclooxygenase-2
IL-6: interleukin-6
IL-1β: interleukin-1 beta
IFN-γ: interferon γ
TNF-α: tumor necrosis factor alpha
Cu/Zn-SOD: copper/zinc SOD
Mn-SOD: manganese SOD
CAT: catalase
HO-1: heme oxygenase-1
Nrf2: nuclear factor E2-related factor 2
NQO1: NAD(P)H quinone dehydrogenase 1
nNOS: neuronal NO synthase
iNOS: inducible NO synthase
NF-κB: nuclear factor kappa-B
eNOS: endothelial nitric oxide synthase.

## References

[1] Rafiee F Mazhari M Ghoreishi M Esmaeilipour O. Effect of lemon verbena powder and vitamin C on performance and immunity of heat-stressed broilers. J Anim Physiol Anim Nutr. (2016) 100:807–12. 10.1111/jpn.12457 26833391

[2] Perdones Á Escriche I Chiralt A Vargas M. Effect of chitosan-lemon essential oil coatings on volatile profile of strawberries during storage. Food Chem. (2015) 197:979–86. 10.1016/j.foodchem.2015.11.054 26617043

[3] Ben Halima N Smaoui S Hamdi N. Citrus lemon essential oil: chemical composition, antioxidant and antimicrobial activities with its preservative effect against *Listeria monocytogenes* inoculated in minced beef meat. Lipids Health Dis. (2017) 16:146. 10.1186/s12944-017-0487-5 28774297 PMC5543433

[4] Lee H Woo M Kim M Noh JS Song YO. Antioxidative and cholesterol-lowering effects of lemon essential oil in hypercholesterolemia-induced rabbits. Prev Nutr Food Sci. (2018) 23:8–14. 10.3746/pnf.2018.23.1.8 29662842 PMC5894780

[5] Ceccarelli I Lariviere WR Fiorenzani P Sacerdote P Aloisi AM. Effects of long-term exposure of lemon essential oil odor on behavioral, hormonal and neuronal parameters in male and female rats. Brain Res. (2004) 1001:78–86. 10.1016/j.brainres.2003.10.063 14972656

[6] Bertuzzi G Tirillini B Angelini P Venanzoni R. Antioxidative action of *Citrus limonum* essential oil on skin. Eur J Med Plants. (2013) 3:1–9. 10.9734/EJMP/2013/1987

[7] Karen W Deborah H Anthony G. A randomised controlled trial of Lavender (*Lavandula angustifolia*) and Lemon Balm (*Melissa officinalis*) essential oils for the treatment of agitated behaviour in older people with and without dementia. Complement Ther Med. (2019) 42:366–73. 10.1016/j.ctim.2018.12.016 30670268

[8] Wrona M Silva F Salafranca J Nerín C Alfonso MJ Caballero MA. Design of new natural antioxidant active packaging: screening flowsheet from pure essential oils and vegetable oils to *ex vivo* testing in meat samples. Food Control. (2021) 120:107536. 10.1016/j.foodcont.2020.107536

[9] Odewumi CO Buggs R Badisa VL Latinwo LM Badisa RB Ikediobi CO. Mitigative action of monoisoamyl-2,3-dimercaptosuccinate (MiADMS) against cadmium-induced damage in cultured rat normal liver cells. Toxicol Vitro. (2011) 25:1733–9. 10.1016/j.tiv.2011.08.013 21911053 PMC3322667

[10] Balogh GT Illés J Székely Z Forrai E Gere A. Effect of different metal ions on the oxidative damage and antioxidant capacity of hyaluronic acid. Arch Biochem Bioph. (2003) 410:76–82. 10.1016/S0003-9861(02)00661-6 12559978

[11] Yosr Z Boussaid M Chograni H Trimech R. Changes in essential oil composition and phenolic fraction in *Rosmarinus officinalis* L. var. typicus Batt. organs during growth and incidence on the antioxidant activity. Ind Crops Prod. (2013) 43:412–9. 10.1016/j.indcrop.2012.07.044

[12] Thomas CJ Callaghan A. The use of garlic (*Alliumsativa*) and lemon peel (*Citrus limon*) extracts as *Culex pipiens* larvacides: persistence and interaction with an organophosphate resistance mechanism. Chemosphere. (1999) 39:2489–96. 10.1016/S0045-6535(99)00161-7

[13] Ferhat MA Meklati BY Chemat F. Comparison of different isolation methods of essential oil from citrus fruits: cold pressing, hydrodistillation and microwave ‘dry' distillation. Flavour Fragrance J. (2007) 22:494–504. 10.1002/ffj.1829

[14] Ferhat MA Boukhatem MN Hazzit M Meklati BY Chemat F. Cold pressing, hydrodistillation and microwave dry distillation of citrus essential oil from algeria: a comparative study. Elect J Biol. (2016) S1:30–41.

[15] Liu Y Liu C Li L. Comparison of vitamin C and its derivative antioxidant activity: evaluated by using density functional theory. ACS Omega. (2020) 5:25467–75. 10.1021/acsomega.0c04318 33043226 PMC7542841

[16] Li GJ Wang J Cheng YJ Tan X Zhai YL Wang Q. Prophylactic effects of polymethoxyflavone-rich orange peel oil on N^ω^-Nitro-*L*-arginine-induced hypertensive rats. Appl Sci. (2018) 8:752. 10.3390/app8050752

[17] Chen Y Wu J Xu Y Fu M Xiao G. Effect of second cooling on the chemical components of essential oils from orange peel (*Citrus sinensis*). J Agri Food Chem. (2014) 62:8786–90. 10.1021/jf501079r 24945493

[18] Li G Tan F Zhang Q Tan A Cheng Y Zhou Q. Protective effects of polymethoxyflavone-rich cold-pressed orange peel oil against ultraviolet B-induced photoaging on mouse skin. J Funct Foods. (2020) 67:103834. 10.1016/j.jff.2020.103834

[19] Warraich UEA Hussain F Kayani HUR. Aging - Oxidative stress, antioxidants and computational modeling. Heliyon. (2020) 6:e04107. 10.1016/j.heliyon.2020.e04107 32509998 PMC7264715

[20] Qian Y Zhang J Fu X Yi R Sun P Zou M. Preventive effect of raw Liubao tea polyphenols on mouse gastric injuries induced by HCl/ethanol *via* anti-oxidative stress. Molecules. (2018) 23:2848. 10.3390/molecules23112848 30388863 PMC6278666

[21] Gedikoglu A Sökmen M Çivit A. Evaluation of *Thymus vulgaris* and *Thymbra spicata* essential oils and plant extracts for chemical composition, antioxidant, and antimicrobial properties. Food Sci Nut. (2019) 7:1704–14. 10.1002/fsn3.1007 31139383 PMC6526640

[22] Caldwell CR. Oxygen radical absorbance capacity of the phenolic compounds in plant extracts fractionated by high-performance liquid chromatography. Anal Biochem. (2001) 293:232–8. 10.1006/abio.2001.5134 11399037

[23] Amiri H. Essential oils composition and antioxidant properties of three thymus species. Evid-based Complement Alternat Med. (2012) 2012:728065. 21876714 10.1155/2012/728065 PMC3163135

[24] Kirkan B Sarikurkcu C Amarowicz R. Composition, and antioxidant and enzyme-inhibition activities, of essential oils from *Satureja thymbra* and *Thymbra spicata* var. spicata. Flavour Fragrance J. (2019) 34:436–42. 10.1002/ffj.3522

[25] Li F Huang G Tan F Yi R Zhou X Mu J. *Lactobacillus plantarum* KSFY06 on D-galactose-induced oxidation and aging in Kunming mice. Food Sci Nutr. (2020) 8:379–89. 10.1002/fsn3.1318 31993164 PMC6977475

[26] Zhu K Zeng X Tan F Li W Li C Song Y. Effect of insect tea on D-galactose-induced oxidation in mice and its mechanisms. Food Sci Nutr. (2019) 7:4105–15. 10.1002/fsn3.1278 31890190 PMC6924339

[27] Zhou Y Tan F Li C Li W Liao W Li Q. White Peony (fermented *Camellia sinensis*) polyphenols help prevent alcoholic liver injury *via* antioxidation. Antioxidants. (2019) 8:524. 10.3390/antiox8110524 31683564 PMC6912415

[28] Pan Y Zhao X Kim SH Kang SA Kim YG Park KY. Anti-inflammatory effects of Beopje curly dock (*Rumex crispus* L.) in LPS-induced RAW 264.7 cells and its active compounds. J Food Biochem. (2020) 44:3291. 10.1111/jfbc.13291 32458452

[29] Willoughby DA Moore AR Colville-Nash PR. COX-1, COX-2, and COX-3 and the future treatment of chronic inflammatory disease. Lancet. (2000) 355:646–8. 10.1016/S0140-6736(99)12031-2 10696997

[30] Chiang YM Lo CP Chen YP Wang SY Yang NS Kuo YH. Ethyl caffeate suppresses NF-kappaB activation and its downstream inflammatory mediators, iNOS, COX-2, and PGE2 *in vitro* or in mouse skin. Br J Pharmacol. (2005) 146:352–63. 10.1038/sj.bjp.0706343 16041399 PMC1576288

[31] Gultekin F Delibas N Yasar S Kilinc I. *In vivo* changes in antioxidant systems and protective role of melatonin and a combination of vitamin C and vitamin E on oxidative damage in erythrocytes induced by chlorpyrifos-ethyl in rats. Arch Toxicol. (2001) 75:88–96. 10.1007/s002040100219 11354911

[32] Apak R Özyürek M Güçlü K Çapanoglu E. Antioxidant activity/capacity measurement. 3. reactive oxygen and nitrogen species (ros/rns) scavenging assays, oxidative stress biomarkers, and chromatographic/chemometric assays. J Agric Food Chem. (2016) 64:1046-1070. 10.1021/acs.jafc.5b04744 26689748

[33] Chen HH Chen YT Huang YW Tsai HJ Kuo CC. 4-Ketopinoresinol, a novel naturally occurring ARE activator, induces the Nrf2/HO-1 axis and protects against oxidative stress-induced cell injury *via* activation of PI3K/AKT signaling. Free Rad Biol Med. (2012) 6:1054–66. 10.1016/j.freeradbiomed.2011.12.012 22245092

[34] Yang CM Lin CC Yang CC Cho RL Hsiao LD. Mevastatin-induced AP-1-dependent HO-1 expression suppresses vascular cell adhesion molecule-1 expression and monocyte adhesion on human pulmonary alveolar epithelial cells challenged with TNF-α. Biomolecules. (2020) 10:381. 10.3390/biom10030381 32121588 PMC7175369

[35] Zhao DR Jiang YS Sun JY Li HH Sun XT Zhao MM. Amelioration of 4-methylguaiacol on LPS-induced inflammation in THP-1 cells through NF-κB/IκBα/AP-1 and Nrf2/HO-1 signaling pathway. J Funct Foods. (2019) 55:95–103. 10.1016/j.jff.2019.01.047

[36] Goven D Boutten A Leçon-Malas V Boczkowski J Bonay M. Prolonged cigarette smoke exposure decreases heme oxygenase-1 and alters Nrf2 and Bach1 expression in human macrophages: roles of the MAP kinases ERK(1/2) and JNK. FEBS Lett. (2009) 583:3508–18. 10.1016/j.febslet.2009.10.010 19822148

[37] Reziwan K Sun D Zhang B Zhao Z. MicroRNA-1225 activates Keap1-Nrf2-HO-1 signalling to inhibit TNFα-induced osteoclastogenesis by mediating ROS generation. Cell Biochem Funct. (2019) 37:256–65. 10.1002/cbf.3394 31017694

[38] Wang X Tang T Zhai M Ge R. Ling-Gui-Zhu-Gan decoction protects H9c2 cells against H_2_O_2_-induced oxidative injury *via* regulation of the Nrf2/Keap1/HO-1 signaling pathway. Evid-based Complement Alternat Med. (2020) 2020:1–11. 10.1155/2020/8860603 33312223 PMC7721500

[39] Yu G Liu X Chen Z Chen H Wang L Wang Z. Ozone therapy could attenuate tubulointerstitial injury in adenine-induced CKD rats by mediating Nrf2 and NF-κB. Iran J Basic Med Sci. (2016) 19:1136–43. 10.22038/ijbms.2016.7740 27872711 PMC5110663

[40] Liu X Chen K Zhu L Liu H Ma T Xu Q. Soyasaponin Ab protects against oxidative stress in HepG2 cells *via* Nrf2/HO-1/NQO1 signaling pathways. J Funct Foods. (2018) 45:110–17. 10.1016/j.jff.2018.03.037

[41] Lacza Z Snipes JA Zhang J Horváth EM Figueroa JP Szabó C. Mitochondrial nitric oxide synthase is not eNOS, nNOS or iNOS. Free Rad Biol Med. (2003) 35:1217–28. 10.1016/S0891-5849(03)00510-0 14607521

[42] You W Wu Z Ye F Wu X. Ginkgolide A protects adverse cardiac remodeling through enhancing antioxidation and nitric oxide utilization in mice with pressure overload. Pharmazie. (2019) 74:698–702. 10.1691/ph.2019.9615 31739841

[43] Miyake Y Murakami A Sugiyama Y Isobe M Koshimizu K Ohigashi H. Identification of Coumarins from lemon fruit (*Citrus limon*) as inhibitors of *in vitro* tumor promotion and superoxide and nitric oxide generation. J Agri Food Chem. (1999) 47:3151–7. 10.1021/jf980999y 10552623

[44] Lin CH Chang CW Wang CC Chang MS Yang LL. Byakangelicol, isolated from *Angelica dahurica*, inhibits both the activity and induction of cyclooxygenase-2 in human pulmonary epithelial cells. J Pharm Pharmacol. (2002) 54:1271–8. 10.1211/002235702320402125 12356282

[45] Choi JS Shin HY Kwon KS Shin S Choung SY Kwon YS. Effects of oxypeucedanin on global gene expression and MAPK signaling pathway in mouse neuroblastoma Neuro-2A cells. Planta Medica. (2011) 77:1512–8. 10.1055/s-0030-1270917 21425034

[46] Li G Cheng Y Zhang T Li Y Han L Liang G. Characterization of oxygenated heterocyclic compounds and *in vitro* antioxidant activity of pomelo essential oil. Drug Des Dev Ther. (2021) 15:937–47. 10.2147/DDDT.S299678 33688168 PMC7936692

[47] Dugo P Russo M. The oxygen heterocyclic components of citrus essential oils. In: Giovanni Dugo LM, editor. Citrus Oils: Composition, Advanced Analytical Techniques, Contaminants, and Biological Activity, Vol. 49. New York, NY: CRC Press (2010). p. 405–44. 10.1201/b10314

